# Automatic gain control of neural coupling during cooperative hand movements

**DOI:** 10.1038/s41598-018-24498-6

**Published:** 2018-04-13

**Authors:** F. A. Thomas, V. Dietz, M. Schrafl-Altermatt

**Affiliations:** 1 Spinal Cord Injury Center, University Hospital Balgrist, 8008, Zürich, Switzerland; 2 Neural Control of Movement Lab, Department of Health Sciences and Technology, ETH Zürich, 8057, Zürich, Switzerland

## Abstract

Cooperative hand movements (e.g. opening a bottle) are controlled by a task-specific neural coupling, reflected in EMG reflex responses contralateral to the stimulation site. In this study the contralateral reflex responses in forearm extensor muscles to ipsilateral ulnar nerve stimulation was analyzed at various resistance and velocities of cooperative hand movements. The size of contralateral reflex responses was closely related to the level of forearm muscle activation required to accomplish the various cooperative hand movement tasks. This indicates an automatic gain control of neural coupling that allows a rapid matching of corrective forces exerted at both sides of an object with the goal ‘two hands one action’.

## Introduction

The neural control of bimanual hand movements is known to be task-and condition-specific^1–7^. Cooperative hand movements, such as opening a bottle, were shown to be task-specifically controlled by a ‚neural coupling’ mechanism^8^. This neural coupling is thought to coordinate the movements between the two hands, i.e. one hand supports the action of the other one. It is task-specifically reflected in the appearance of EMG reflex responses in the activated forearm muscles of both sides to unilateral arm nerve stimulation, while during bimanual non-cooperative hand movements only ipsilateral reflex responses appear^8^. This observation indicates an involvement of both ipsi-and contralateral hemispheres in the control of cooperative hand movements. Hitherto the neural coupling mechanism was investigated using a standard protocol, i.e. movement speed 0.75 Hz and 20% maximal voluntary contraction (MVC). The aim of this study was to evaluate the effect of varying movement resistance and velocity on the neural coupling.

From earlier studies it is known that the amplitude of reflex activity ipsilateral to the site of stimulation depends on the level of background muscle activity of the muscle that becomes perturbed by stretching^9–12^ or by electrical arm nerve stimulation^13^. This behavior was interpreted as an automatic servo action to rapidly compensate for movement perturbations. In this study the perturbation induced during cooperative hand movements does not consist in a muscle stretch but in a non-noxious arm nerve stimulation with the focus directed to the reflex behavior contralateral to the site of stimulation. It is hypothesized that the behavior of contralateral reflex responses is coupled to that of the ipsilateral ones in order to match the forces exerted at the object between the two sides, i.e. a more demanding movement condition might lead to a stronger neural coupling.

## Results

**Figure 1:**
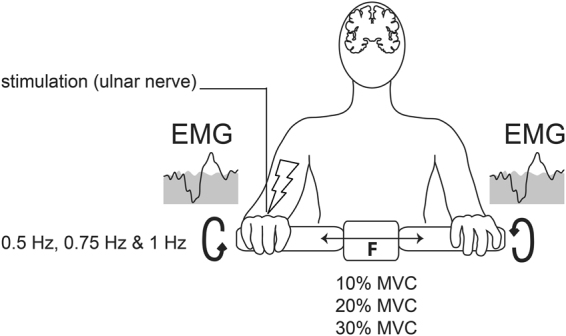
Experimental setup and device used. Electrical stimulations were applied during cooperative hand movements with different movement demands (i.e. three wrist extension/flexion frequencies against three resistances). The handles of the device used are mechanically coupled i.e. during cooperative hand movements the torque produced from one limb has to be counteracted by the other limb.

**Figure 2:**
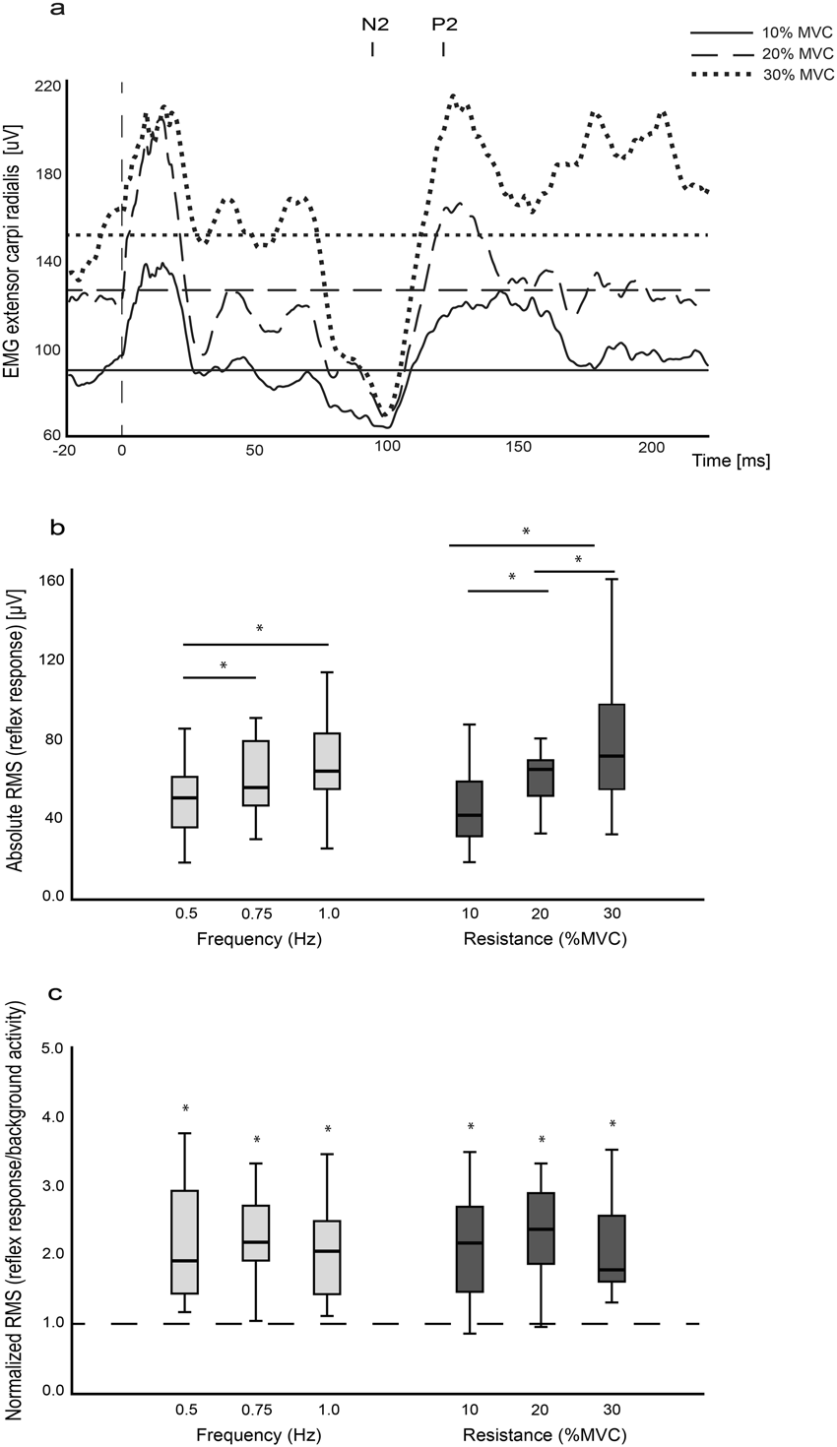
Influence on the contralateral EMG reflex response from different movement conditions. (**a**) Grand averages of the contralateral EMG reflex responses at 0.75 Hz movement velocity against three resistances from all subjects (n = 15). The horizontal lines indicate the levels of pre-stimulus muscle activity of the forearm extensor. The vertical dashed line indicates the timepoint of stimulation. N2 and P2 represent the negative and positive components of the contralateral reflex response (see methods); (**b**) Absolute contralateral EMG reflex response amplitudes from all subjects (given as RMS). Asterisks indicate significant differences between conditions; (**c**) Contralateral EMG reflex responses normalized to prestimulus muscle activity (horizontal dashed line) from all subjects. Asterisks indicate significant differences between reflex response and prestimulus muscle activity. In (**b** and **c**), boxes represent the interquartile range (25^th^–75^th^ percentile) separated by the median. Outliers were removed from the box-plots for illustration purpose.

Data was analyzed from all fifteen subjects. All subjects were able to perform the nine movement conditions (i.e. each movement velocity was performed against each movement resistance, Fig. 1). The analysis of reflex responses was focused on the forearm extensor muscles contralateral to the stimulation site as a marker for the neural coupling mechanism. Distinct reflex responses were present in the forearm muscles contralateral to the site of nerve stimulation during all conditions. Wilcoxon signed ranked tests revealed that RMS values of the reflex response differed significantly from those of the background EMG in all conditions (all *p* < 0.01). Figure 2a shows the relationship between reflex amplitudes and level of background EMG. The grand averages of the contralateral reflex responses are shown during a movement frequency of 0.75 Hz at three resistances. The plot shows that the stronger the level of background activity was, the larger were the reflex response amplitudes. Figure 2b shows the box plot of the absolute RMS values of the reflex responses grouped for the three movement velocities/frequencies and resistances. The reflex amplitude increased significantly from 0.5 Hz (51.2, 36.2–61.8 µV) to 0.75 Hz (56.4, 47.3–79.8 µV) (*t*(14) = −3.69, *p* = 0.014) and from 0.5 Hz to 1 Hz (64.7, 55.7–83.6 µV) (*t*(14) = 3.71, *p* = 0.014). The RMS of the reflex responses grouped for the three resistances increased significantly from 10% (42.4, 31.7–59.5 µV) to 20% MVC (65.6, 52.2–70.3 µV) (*t*(14) = −3.69, *p* = 0.014), from 10% to 30% (*t*(14) = −5.43, *p* = 0.0005) and from 20% to 30% MVC (72.4, 55.5–98.3 µV) (*t*(14) = −4.04, *p* = 0.007). When the reflex amplitudes were normalized to the background activity, Friedman tests revealed no significant difference between the resistance (Chi^2^(2) = 1.733, *p* = 0.42) or velocity (Chi^2^(2) = 0.133, *p* = 0.93) conditions (Fig. 2c).

**Figure 3:**
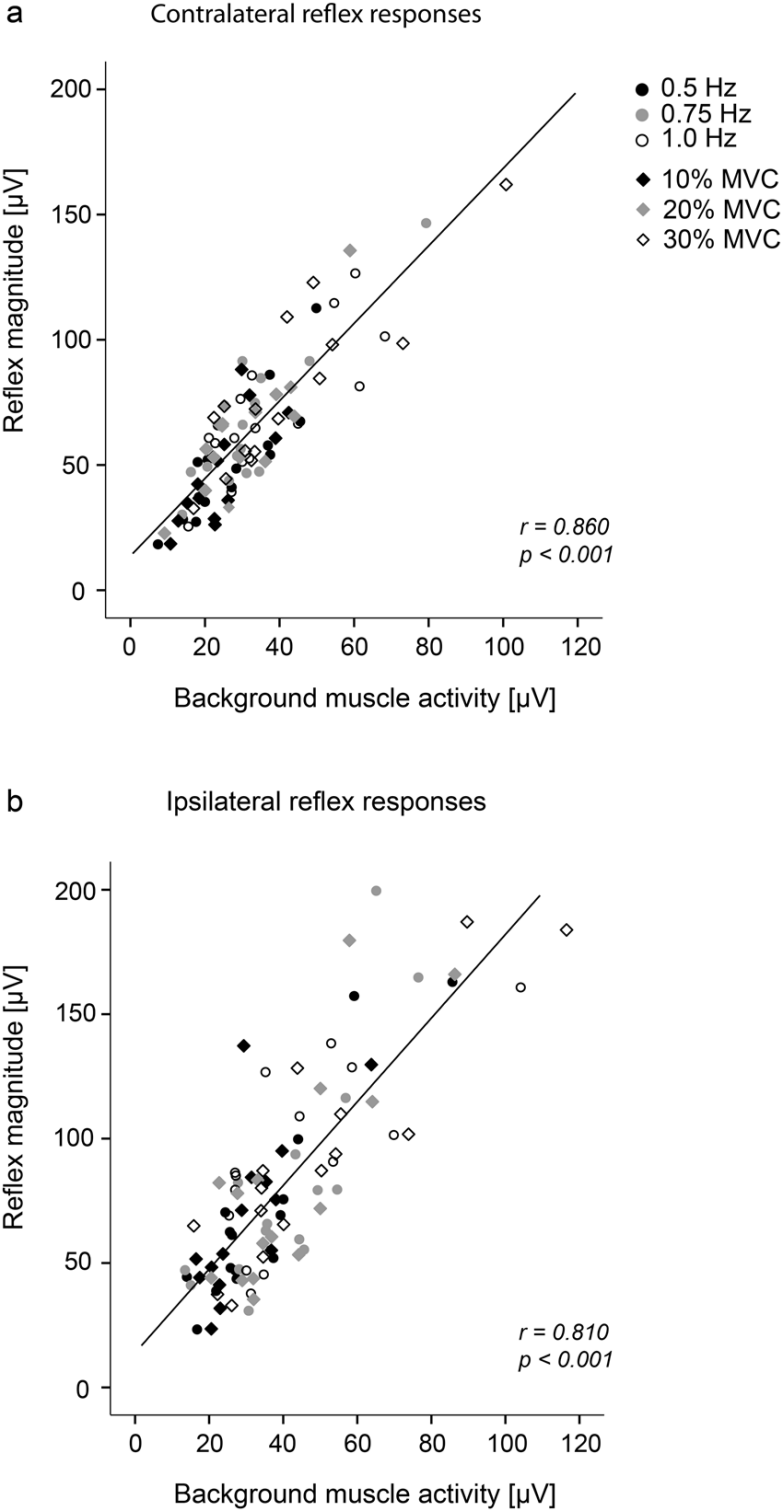
Relationship between the level of background muscle activity [µV] and the reflex magnitude [µV] in the extensor muscle for all subjects (n = 15) (**a**) contralateral and (**b**) ipsilateral to the side of stimulation. Samples were grouped according to the movement conditions ‘velocity’ and ‘resistance’ (see Methods section). Both plots show a strong linear correlation i.e. the higher the level of background muscle activity, the higher the corresponding reflex response.

Pearson correlation coefficient revealed a strong correlation between the level of background muscle activity and the magnitude of the reflex response for both contralateral (r = 0.860, p > 0.001, Fig. 3a) and ipsilateral (r = 0.810, p > 0.001, Fig. 3b) reflex responses.

The mean latency of the contralateral EMG reflex responses across all movement conditions amounted to 88.7 (80.7–93.3 ms). There was no significant difference in latency between the different conditions.

## Discussion

The aim of this study was to explore the influence of movement velocity (i.e. change in frequency) and resistance during cooperative hand movements on the neural coupling mechanism. This neural coupling is reflected in the task-specific appearance of reflex EMG responses (i.e. not present during separate non-cooperative movements) in forearm muscles contralateral to the site of stimulation^8^.

The main result obtained were, 1. Contralateral reflex responses appeared in all movement conditions even at slow velocity and low resistance; 2. The increase in size of contralateral reflex responses paralleled the level of forearm muscle activity associated with higher movement velocities and resistances, i.e. the ratio of reflex response amplitude to background EMG amplitude remained constant.

It has been shown for cyclic movements of the upper limb that cutaneous reflexes are modulated depending on the movement phase^14^. Randomly released stimuli might therefore lead to non-standardized reflex magnitudes within a movement condition. However, averaging all EMG responses within one condition will minimize a possible bias in reflex magnitude related to different movement phases. This issue is further compensated by normalizing the reflex magnitude to pre-stimulus muscle activity (instead of normalization to unstimulated (dummy) EMG within the same time window; see section “EMG recordings”).

In earlier studies on the behavior of reflex responses a dependency of the ipsilateral reflex response on the intensity of mechanical stimulation was thought to compensate for limb disturbance^9–11^ (for review^15^). Later on, the appearance of reflex responses not only in the perturbed limb but also in non-stimulated, synergistically acting limb muscles was described to occur during functional movements such as locomotion^16^, arm cycling^14^ or cooperative hand movements^8^. In these studies not mechanical stimuli but nerve stimulation was used to induce limb pertubations. The present study shows that the contralateral reflex response amplitude to unilateral nerve stimulation automatically increases with the level of background EMG, i.e. with the effort exerted by the hands, produced by muscles of both forearms involved in the performance of the cooperative task. Such an increase of reflex gain with the level of muscle activation was hitherto reported only for perturbing the ipsilateral thumb muscle^10^. An automatic gain scaling of short latency spinal reflexes was described^12^. Such a behavior can hardly be expected to occur in forearm muscles contralateral to the site of stimulation. Here we can show for the first time that such an automatic gain scaling of long-latency reflex activity occurs contralateral to the site of stimulation.

It is suggested that this reflex behavior reflects the functional significance of the neural coupling. By this mechanism, the level of forearm muscle activation, required for an effective performance of the various cooperative hand movement tasks becomes matched between the two sides, i.e. the reciprocal forces acting on an object have to be adjusted to a level that is needed to overcome the resistance and to rapidly compensate any perturbation (e.g. unilateral nerve stimulation) simultaneously on both sides. Any difference in effort produced between the hands would not allow the successful performance of the task, e.g. to open a bottle. This observation fits with the idea of an ‚automatic gain scaling’ or ‘automatic servo action’ of reflex behavior^9–12^. Based on the present study this reflex behavior on the ipsilateral stimulated side can now be extended to the contralateral cooperative but not perturbed hand/arm. It has to remain open what exact pathways are mediating the automatic adjustments. Nevertheless it is obvious that ipsi-as well as contralateral hemispheres have to be involved in the neural coupling mechanism.

The observations made here support the idea of a ‘two hands—one action’ mechanism. However, it has to remain open in how far this idea can be generalized to more complex movements requiring unequal contributions of both hands for a unified action. In conclusion it could be shown that during cooperative hand movements an automatic scaling of reflex activity does not only take place ipsilateral^10,11^ but also contralateral to the site of stimulation.

## Methods

This study was approved by the Cantonal Ethic Commission of Zürich, subcommittee for orthopaedics and locomotor system, and conformed to the standards set by the declaration of Helsinki. All subjects were informed about the experiment and had to give written consent before any measurements were conducted.

### Experimental protocol

EMG reflex responses to unilateral right ulnar nerve stimulation were recorded in forearm extensor and flexor muscles of both sides (Fig. 1) during cooperative hand movements in fifteen healthy subjects (age: 27.0 ± 6.2 years; 10 female/5 male). For the cooperative movement tasks a device was used that allowed counteractive rotations of handles, similar to that described previously^8,17^. With this device, movements were performed with rhythmic alternating antiphase wrist extension and flexion mimicking a “bottle opening” task (Fig. 1). For different movement conditions, three velocities (0.5 Hz, 0.75 Hz and 1 Hz,i.e, one flexion/extension cycle lasted for about 2 s, 1,33 s or 1 s, respectively) and three resistances (10%, 20% and 30% of maximal voluntary contraction(MVC)) were chosen. Every resistance condition was performed at each of the three movement frequencies resulting in a total of nine conditions. Subjects performed every condition once in a randomized order. MVC was determined as the highest value of three maximal isometric wrist extension movements of the non-dominant arm. A mechanical break between the handles of our device allowed to change the resistance of cooperative movements. An implemented force sensor provided the control of the resistance exerted by the break. Thus, the resistance could be exactly set to the individual %MVC for each subject and for every condition. Frequencies were indicated by a metronome.

### Electrical nerve stimulation

The ulnar nerve of the right arm was stimulated with a Keypoint Focus (Natus®, Pleasanton, USA) through self-adhesive surface electrodes (Ambu® A/S Neuroline 700, Denmark) 10 times every 3–8 s during each of the conditions. The movement condition in previous studies (i.e. 20% MVC with 0.75 Hz frequency^8,18^) allowed for 30 stimulations (i.e. 15 per side) while the condition 1 Hz frequency against 30%MVC in the present protocol is difficult to maintain for a similar duration. Thus, we reduced the number of stimulations to 10 in order to prevent fatigue and to maintain a standardized movement execution throughout every condition. The stimulation electrodes (inter-electrode distance 2 cm, cathode proximal) were placed just proximal to the wrist crease. Stimulations were triggered randomly within the movement cycles. Stimulations were timed to the onset of the movement cycle in previous studies. We used a slightly different device in the present study where automatic triggering of a stimulation related to a specific position was not possible. Stimulation intensity (SI) was set at 150% above motor threshold (MT - first visible twitch of the abductor digiti minimi). Stimulations consisted of a 333 Hz train of four biphasic pulses of 1 ms duration resulting in a total stimulus duration of 10 ms. There are two factors which have determined the number of executed movement cycles namely the movement frequency of the condition (0.5 Hz, 0.75 Hz or 1 Hz) and the stimulation frequency (variation between 3 and 8 seconds). Ten stimulations were applied in each condition. Given an example frequency of 1 Hz (i.e. 1 movement cycle/s), participants performed between 30–80 movement cycles (depending on the stimulations) for this condition.

### EMG recordings

EMG activity of wrist extensor (extensor carpi radialis) and flexor (flexor carpi ulnaris) muscles of both forearms was recorded (Noraxon, Scottsdale, AZ, USA) using two single hydrogel knob surface electrodes (Kendall^TM^ H124SG, 2,4 cm diameter), sampled (1500 Hz), band-pass filtered (10–10.000 Hz) and post-processed as previously described^18^. The root mean square (RMS) of the rectified signal in the time window between 75 ms and 135 ms after stimulation onset was calculated including the main components (i.e. N2, P2) of the reflex response. The RMS of the rectified reflex response was normalized to the background activity −30 ms to −10 ms pre-stimulation. Different levels of MVC between the subjects resulted in heterogeneous levels of the corresponding background EMG in the different conditions. Therefore a normalization procedure of background EMG was performed for every subject before the descriptive analysis (e.g. grand average) of the data. The absolute and the normalized RMS values of the reflex responses were grouped for the different movement conditions (i.e. for each velocity condition the mean values of the reflex responses obtained during the movements against three resistances were averaged, and vice versa, for each resistance condition the mean values of the reflex responses obtained during the three velocities were averaged).

### Statistical analysis

All statistical procedures were performed using the SPSS version 23 (IBM® Statistics). To test whether a contralateral reflex response was evoked, absolute EMG RMS values of every condition and the grouped RMS values were tested with a two-sided Wilcoxon signed rank test in relation to the corresponding background RMS. One-way repeated measures ANOVAs were used to compare the grouped absolute RMS reflex responses between the movement conditions. Paired two-sided T-Tests were chosen as post-hoc tests. Grouped normalized RMS values were not normally distributed, thus, Friedman tests were used to detect possible between the different movement conditions. The correlation between background muscle activity and reflex magnitude was calculated with Pearson correlation for grouped ipsilateral and contralateral responses. In all tests, p-values lower than 0.05 were considered as significant. All tests were corrected for multiple comparisons with a Bonferroni correction. If not stated otherwise, absolute and normalized RMS values are given as median and interquartile range (IQR, 25^th^–75^th^ percentile).

### Data availability statement

The datasets generated during and/or analyzed during the current study are available from the corresponding author on reasonable request.
